# Two-dimensional gel electrophoresis data in support of leaf comparative proteomics of two citrus species differing in boron-tolerance

**DOI:** 10.1016/j.dib.2015.04.018

**Published:** 2015-05-05

**Authors:** Wen Sang, Zeng-Rong Huang, Yi-Ping Qi, Lin-Tong Yang, Peng Guo, Li-Song Chen

**Affiliations:** a Institute of Horticultural Plant Physiology, Biochemistry and Molecular Biology, Fujian Agriculture and Forestry University, Fuzhou 350002, China; b College of Resources and Environmental Sciences, Fujian Agriculture and Forestry University, Fuzhou 350002, China; c Institute of Materia Medica, Fujian Academy of Medical Sciences, Fuzhou 350001, China; d The Higher Educational Key Laboratory of Fujian Province for Soil Ecosystem Health and Regulation, Fujian Agriculture and Forestry University, Fuzhou 350002, China; e Fujian Key Laboratory for Plant Molecular and Cell Biology, Fujian Agriculture and Forestry University, Fuzhou 350002, China

**Keywords:** Citrus, Boron-toxicity, Proteomics, Two-dimensional gel electrophoresis (2-DE)

## Abstract

Here, we provide the data from a comparative proteomics approach used to investigate the response of boron (B)-tolerant ‘Xuegan’ (*Citrus sinensis*) and B-intolerant ‘Sour pummelo’ (*Citrus grandis*) leaves to B-toxicity. Using two-dimensional gel electrophoresis (2-DE) technique, we identified 50 and 45 protein species with a fold change of more than 1.5 and a *P*-value of less than 0.05 from B-toxic *C. sinensis* and *C. grandis* leaves. These B-toxicity-responsive protein species were mainly involved in carbohydrate and energy metabolism, antioxidation and detoxification, stress responses, coenzyme biosynthesis, protein and amino acid metabolism, signal transduction, cell transport, cytoskeleton, nucleotide metabolism, and cell cycle and DNA processing. A detailed analysis of this data may be obtained from Sang et al. (J. Proteomics 114 (2015))[1].

**Specifications table**

**Table:** 

Subject area	Biology

More specific subject area	Boron (B)-toxicity-induced protein changes in citrus leaves
Type of data	Protein abundances
How data was acquired	Two-dimensional gel electrophoresis (2-DE) and mass spectrometry
Data format	Normalized data
Experimental factors	Different citrus species (B-tolerant *Citrus sinensis* and B-intoerant *Citrus grandis*), B-toxicity
Experimental features	Citrus seedlings were treated with excess B and leaf proteome were separated using 2-DE. Differentially abundant protein species in B-toxic and control leaves were identified using MALDI-TOF/TOF-MS analysis.
Data source location	Fujian Agriculture and Forestry University, Fuzhou, China
Data accessibility	The data are with this article

**Value of data**

• Data provided B-toxicity-induced changes in leaf protein profiles of two citrus differing in B-tolerance.
• B-toxicity-responsive protein species in leaves differed between the two citrus species.
• The integrated proteomic analysis provided a better insight into the tolerant mechanism of citrus plants to B-toxicity at the translational level.

## 1 Data, experimental design, materials and methods

Boron (B)-tolerant ‘Xuegan’ (*Citrus sinensis*) and B-intolerant ‘Sour pummelo’ (*Citrus grandis*) seedlings were used to investigate the effects of B-toxicity on leaf proteomics. Plant culture and B treatments were performed as described previously [1,2]. Briefly, 13 week-old seedlings of *C. sinensis* and *C. grandis* grown in 6 L pots (two seedlings per pot) containing river sand in a greenhouse under natural photoperiod were irrigated every other day until dripping with nutrient solution containing 10 μM (control) or 400 μM (B-toxic) H_3_BO_3_ for 15 weeks. There were 20 pots per treatment in a completely randomized design. At the end of the experiment, fully expanded leaves collected from B-toxic and control plants at noon under full sun were immediately frozen in liquid N_2_. Frozen samples were stored at −80 °C until extraction.

## 2 Protein extraction

About 1 g leaves collected equally from five different replicates (one plant per replicate, one leaf per plant) were mixed as a biological replicate. There were three biological replicates for each treatment. Proteins from control and B-toxic frozen leaves were extracted according to Yang et al. [3] using a phenol extraction procedure.

## 3 2-Dimensional gel electrophoresis (2-DE)

2-DE was performed according to You et al. [4]. Gel image analysis was performed using PDQuest software (version 8.0.1, Bio-Rad, Hercules, CA, USA). Spot intensities were subjected to statistical analysis to obtain the differentially abundant protein spots. A protein spot was considered differentially abundant between samples when it had both a *P*-value of less than 0.05 and a fold change of more than 1.5. Based on the two criteria, we obtained 32 up-regulated and 28 down-regulated protein spots from B-toxic *C. sinensis* leaves, and 31 upregulated and 31 down-regulated protein spots from B-toxic *C. grandis* leaves. All these differentially abundant protein spots in B-toxic *C. sinensis* and *C. grandis* leaves are presented in Supplementary Tables 1 and 2.

## 4 Identification of differentially abundant protein spots using mass spectrometry

All theses differentially abundant protein spots were excised from the colloidal Coomassie Brilliant Blue stained gels and subjected to MS analysis, which was performed on an AB SCIEX 5800 TOF/TOF. All acquired spectra of samples were processed using TOF/TOF ExplorerTM Software (AB SCIEX) in a default mode. The data were searched by GPS Explorer (Version 3.6) with the search engine MASCOT (Version 2.3, Matrix Science Inc, Boston, MA). Protein spots with scores greater than 75 were considered statistically significant (*P*<0.05) and accepted. Bioinformatics analysis of proteins was performed according to Yang et al. [3]. Here, we identified 50 and 45 differentially abundant protein species from B-toxic *C. sinensis* and *C. grandis* leaves. A detailed data could be obtained from Supplementary Tables 3 and 4.

## 5 qRT-PCR analysis of genes for some differentially abundant protein species

To understand the correlation between gene and protein expression levels, we used qRT-PCR to assay the transcript levels of genes encoding 14 differentially expressed protein species in B-toxic *C. sinensis* and *C. grandis* leaves (Supplementary Table 5). The transcript levels of all these genes were in agreement with our 2-DE data except for genes encoding glutathione *S*-transferase (GST) GST1 (S17), ribulose bisphosphate carboxylase activase (S39) and ribosomal protein L12 (G50), demonstrating that most of these differentially abundant protein species were regulated in the transcriptional level.
